# ERP correlates of word production predictors in picture naming: a trial by trial multiple regression analysis from stimulus onset to response

**DOI:** 10.3389/fnins.2014.00390

**Published:** 2014-12-04

**Authors:** Andrea Valente, Audrey Bürki, Marina Laganaro

**Affiliations:** Faculté de Psychologie et des Sciences de l'Éducation, University of GenevaGeneva, Switzerland

**Keywords:** ERP, topographies, single trial, picture naming, encoding processes

## Abstract

A major effort in cognitive neuroscience of language is to define the temporal and spatial characteristics of the core cognitive processes involved in word production. One approach consists in studying the effects of linguistic and pre-linguistic variables in picture naming tasks. So far, studies have analyzed event-related potentials (ERPs) during word production by examining one or two variables with factorial designs. Here we extended this approach by investigating simultaneously the effects of multiple theoretical relevant predictors in a picture naming task. High density EEG was recorded on 31 participants during overt naming of 100 pictures. ERPs were extracted on a trial by trial basis from picture onset to 100 ms before the onset of articulation. Mixed-effects regression models were conducted to examine which variables affected production latencies and the duration of periods of stable electrophysiological patterns (topographic maps). Results revealed an effect of a pre-linguistic variable, visual complexity, on an early period of stable electric field at scalp, from 140 to 180 ms after picture presentation, a result consistent with the proposal that this time period is associated with visual object recognition processes. Three other variables, word Age of Acquisition, Name Agreement, and Image Agreement influenced response latencies and modulated ERPs from ~380 ms to the end of the analyzed period. These results demonstrate that a topographic analysis fitted into the single trial ERPs and covering the entire processing period allows one to associate the cost generated by psycholinguistic variables to the duration of specific stable electrophysiological processes and to pinpoint the precise time-course of multiple word production predictors at once.

## Introduction

The representations and processes underlying word processing for speech production have been studied extensively for more than three decades with various experimental approaches, including the analysis of speech errors (e.g., Fromkin, 1971; Garrett, 1980; Dell, 1990), chronometric paradigms (Bock, 1996), eye movements studies (e.g., Griffin, 2001), and event-related potential (ERP) approaches (Ganushchak et al., 2011). In language production research, one of the main endeavors has been to unveil the properties and time course of the encoding stages involved in producing a word. Most chronometric studies involve picture naming tasks where the dependent variable is the time interval between picture presentation and the onset of articulation (see Johnson et al., 1996 for a review). In many of these picture naming experiments, the properties of the words (e.g., frequency, age of acquisition, length) or of the pictures (e.g., visual complexity) are manipulated. On the basis of the influence that such properties exert on picture naming latencies relative to their influence on response times in other tasks (e.g., word-picture matching, Jescheniak and Levelt, 1994), inferences are drawn on the organization of words in memory and/or on the processes underlying their production. Within the framework of chronometric approaches, eye movements studies have provided information on the relation between gaze and the planning and execution of utterances, allowing to pinpoint the time course of the encoding stages involved in word production (e.g., Meyer et al., 1998; Griffin, 2001).

More recently, ERP studies have begun to examine which time periods are modulated by specific psycholinguistic variables, in order to associate these effects with the time course of underlying encoding processes. Both approaches have specific limitations due to methodological constraints. On the one hand, behavioral chronometric methods allow the investigation of several relevant variables simultaneously, but the precise time course of their effects can only be inferred by summoning the results of different studies (e.g., Alario et al., 2004). On the other hand, ERP studies allow insight into the time periods affected by specific variables, but usually investigated a few variables at a time (e.g., Cheng et al., 2010; Strijkers et al., 2010).

A more indirect contribution to this debate is provided by studies associating behavioral techniques and functional magnetic resonance imaging (fMRI) with the purpose of mapping the neural substrates of the processing stages involved in word production (e.g., Graves et al., 2007; Wilson et al., 2009). However, fMRI technique lacks the precise temporal resolution provided by EEG and ERPs.

The present research provides a novel and complementary approach by investigating simultaneously the effects of multiple theoretically relevant psycholinguistic variables on ERPs covering the entire word encoding period from picture onset to articulation. We expect that this approach will provide new information on the temporal characterization of the encoding stages involved in word production from presentation of the picture to the articulation of the corresponding word.

In the following section we will briefly review the psycholinguistic approaches and ERP studies that have examined the time course of word encoding, before describing the approach of the present study.

Models of word production agree on the fact that speakers have to go through a sequence of three major cognitive processes before they can articulate the name corresponding to a picture (e.g., Glaser, 1992; Levelt et al., 1999), although different claims are made regarding the dynamics of these encoding processes (Dell, 1986, 1988). The first process involves visual processing and leads to object recognition. The second process involves the activation of the corresponding concept. It is only at the third processing stage that language gets involved, with the encoding of the corresponding word. This step, often referred to as the formulation process (e.g., Levelt et al., 1999) has been extensively detailed in the psycholinguistic literature and is assumed to entail several processing sub-stages: lexical selection, phonological encoding, and phonetic encoding. Lexical selection corresponds to the retrieval from the mental lexicon of a lemma, i.e., a semantically and syntactically specified representation (lexical-semantic processes). The word's phonological representation or lexeme is specified during phonological encoding (lexical-phonological processes); then, on the basis of the abstract phonological codes, syllable-sized articulatory gestures and their temporal relationships are either computed or retrieved (phonetic encoding) before articulation can start. The average time needed to start articulating a word from picture onset is less than a second. More recently, a major effort has been devoted to characterizing the precise time course of these processes, that is, their respective order and duration. As is evident from previous reviews (Indefrey and Levelt, 2004; Indefrey, 2011) this issue is particularly complex and our current knowledge, which relies on the comparison of disparate sources of evidence, is still incomplete.

Information about when and how the different encoding processes unfold can be extracted from different sources (see Indefrey and Levelt, 2004 for a comprehensive review). A first way to obtain such information is to design paradigms that target specific processes. However, these approaches do not allow estimating directly the time course of specific processes. For instance, Jescheniak and Levelt (1994) had participants perform a picture word matching task and subtracted an approximation of the time devoted to response preparation and execution from the overall response times to conclude that it takes less than 150 ms to access lexical concepts. Another approach to gain insight into the time course of word production processes is the use of priming or interference paradigms where the prime (an auditory or visually presented word distractor) occurs at different time points relative to picture presentation (or SOA, for *stimulus onset asynchrony* e.g., Glaser and Düngelhoff, 1984). Distractors typically have a phonological, semantic or sometimes syntactic relationship with the target word. Depending on the SOA at which a given distractor type affects responses, conclusions have been drawn on the temporal relationship between specific encoding processes (e.g., Schriefers et al., 1990), while not necessarily on their precise time course.

A third important source of information on the time course of cognitive processes comes from EEG or MEG studies with ERPs, which allow one to track temporal information with a precision at the millisecond range. Different paradigms have been used so far, including delayed picture naming tasks (Jescheniak et al., 2003; Cornelissen et al., 2004; Vihla et al., 2006; Laganaro et al., 2009), implicit naming or metalinguistic tasks (e.g., Thorpe et al., 1996; Van Turennout et al., 1998; Schmitt et al., 2000; Jescheniak et al., 2002; Rodriguez-Fornells et al., 2002; Zhang and Damian, 2009) and, more recently, overt picture naming (see Ganushchak et al., 2011; Strijkers and Costa, 2011 for a critical review of EEG/MEG speech production studies). ERP paradigms using overt picture naming paradigms are the most relevant as they truly involve an overt production of the target words. Studies conducted so far with this task have addressed one single step or sub-step of the production process each. They have usually involved a manipulation of the experimental conditions (e.g., semantic context, Costa et al., 2009; Aristei et al., 2011; Blackford et al., 2012) or of the materials, using factorial or semi-factorial designs, i.e., with two subsets of items varying in terms of a specific predictor being compared (e.g., name agreement, Cheng et al., 2010; age of acquisition, Laganaro and Perret, 2011; lexical frequency, Levelt et al., 1998; Strijkers et al., 2010).

In the present study, we extend this second approach, by considering most variables described in previous chronometric and ERP studies at once.

A similar methodological approach involving multiple regression analyses between ERPs and psycholinguistic factors was first introduced by Hauk et al. (2006) in a visual word recognition paradigm. In their study the authors orthogonalized four theoretically relevant psycholinguistic factors in visual word recognition and investigated their effects on ERP regression coefficients, with the aim of determining which factors affected neurophysiological activity, and to obtain information on the precise time course of their effects during word processing. Dien et al. (2003) also introduced a novel ERP approach aimed at avoiding the grouping of experimental stimuli in few categories and the potential subsequent loss of information. This was achieved by averaging the items across participants, rather than across trials, in order to investigate correlations between stimulus characteristics and the neurophysiological activity.

In relation to these previous studies, here the analyses of the effects of psycholinguistic variables on neurophysiological activity are carried out on the duration of periods of stable global electrophysiological activity on the whole word encoding process from picture onset to articulation. This allows to determine the origin of the cost generated by these variables on vocal response times and to assess the effect exerted by psycholinguistic factors on the different stages of information processing.

With respect to Dien et al. (2003), the item-averaging approach was further improved insofar as here ERP trials were not averaged and template maps issued from the spatio-temporal segmentation of group-averaged ERPs were backfitted in single trials. Single trial approaches have proved reliable and effective as they allow preserving the complete variability of the EEG dataset, which is usually lost when averaged responses are utilized (e.g., De Lucia et al., 2007, 2010). Thus, our approach also extends the classical topographic analysis in fitting the spatio-temporal segmentation of the group-averaged ERPs back into the single trials rather than into the subject-averaged ERPs.

This analysis is likely to inform us on two different issues. Firstly, ERP modulations by variables that can be unambiguously attributed to given word encoding processes will provide precise information on the time course of these specific processes. Secondly, if effects are found for variables whose attribution still lacks empirical support, our findings, together with existing estimates of the time course of the production process, will allow us to propose a specific locus for these variables.

**Figure 1:**
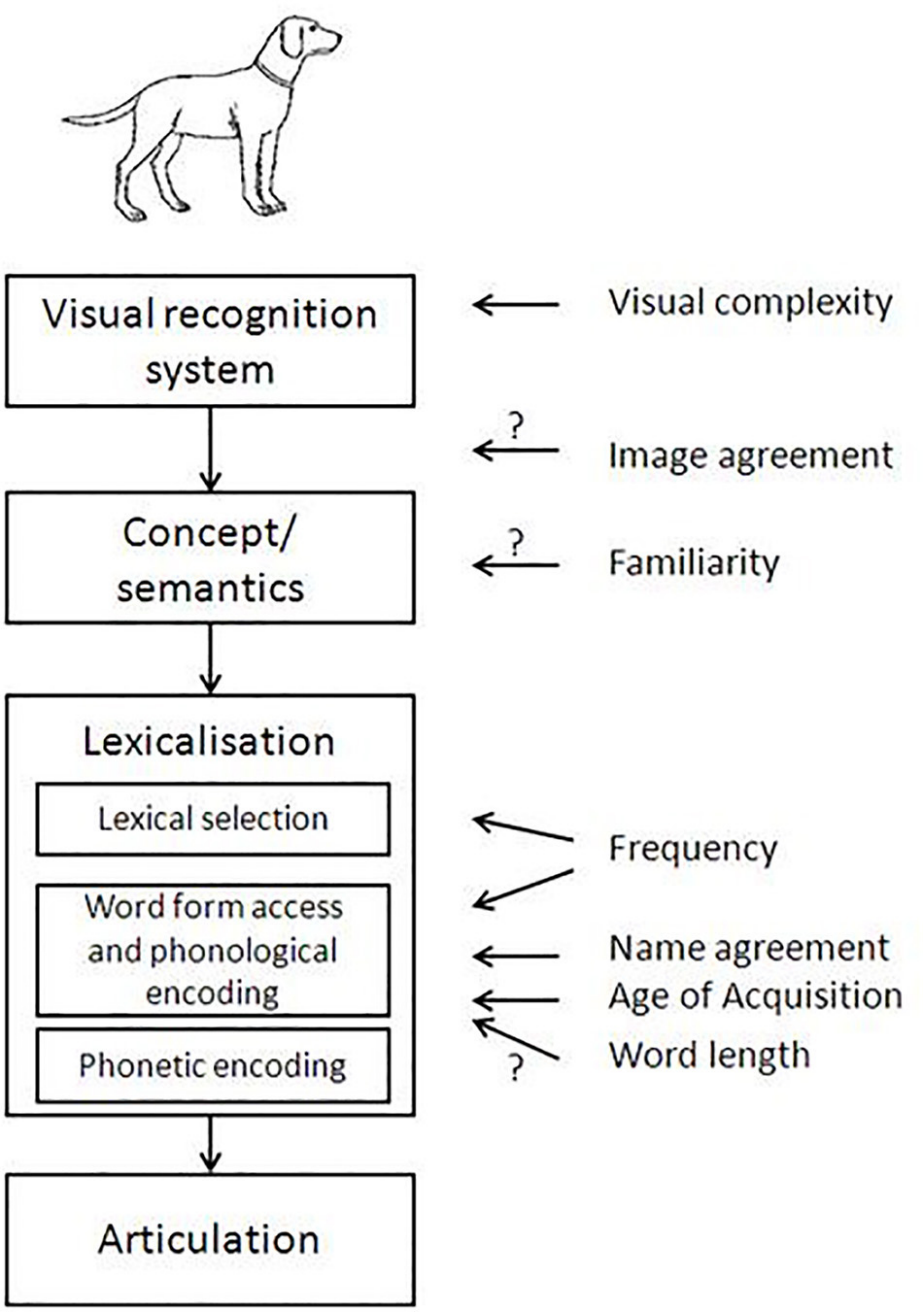
**Picture naming model adapted from Alario et al. (2004), with an indication of the psycholinguistic factors exerting an effect on each specific encoding substage (according to the literature review)**. We have adapted this figure to include more variables and clarify whether the locus is supported by empirical data or not.

Based on the existing literature, the following variables were included in our analysis: Visual complexity, Concept familiarity, Image agreement, Name agreement, Lexical frequency, Age of acquisition, Word length, Phonological neighborhood, and Phonotactic probability. Figure 1 shows these variables and the processing level with which they have been associated in previous studies. Further details on each variable are provided below.

*Visual complexity*, defined as “*the amount of detail or intricacy of line in a picture*” (Snodgrass and Vanderwart, 1980), has been associated with object recognition. Empirical evidence in favor of this insofar intuitive hypothesis has recently been found by Martinovic et al. (2008). The authors reported that the visual complexity of line drawings modulated waveforms in the P1 range, a time window likely associated with visual processes and object recognition. Note also that whereas a few studies found increasing response latencies for more complex pictures (Attneave, 1957; Alario et al., 2004), other studies failed to find differences in response latencies between high and low complexity pictures (Paivio et al., 1989; Snodgrass and Yuditsky, 1996; Barry et al., 1997; Cuetos et al., 1999; Bonin et al., 2002, 2003; Janssen et al., 2011) or reported the opposite effect, i.e., decreased production latencies for more complex pictures (Szekely et al., 2005).

*Concept familiarity* is defined as “*the degree to which participants come in contact with or think about the concept*” (Snodgrass and Vanderwart, 1980, p. 183). It has been estimated by asking participants to rate on a five-point scale (from 1. *very unfamiliar* to 5. *very familiar* object), the extent to which the concept associated with a picture was recurrent in their thoughts or frequently encountered (Alario and Ferrand, 1999). Concept familiarity has been hypothesized to affect the links between picture representations and their semantic representations (Hirsh and Funnell, 1995). To our knowledge, however, this hypothesis has not been confirmed empirically. As for the previous variable, effects of concept familiarity on picture naming latencies have not been reported systematically. A few studies found increasing response latencies for less familiar pictures (Snodgrass and Yuditsky, 1996; Ellis and Morrison, 1998), while other studies failed to find differences in response latencies between items of high and low familiarity (Barry et al., 1997; Dell'Acqua et al., 2000; Bonin et al., 2002, 2003; Alario et al., 2004).

*Lexical frequency* refers to how often the word is used in a language. Shorter response latencies for more frequent words have been reported many times in the psycholinguistic literature (e.g., Jescheniak and Levelt, 1994; Barry et al., 1997; Ellis and Morrison, 1998; Griffin and Bock, 1998; Alario et al., 2004). However the effect of lexical frequency is mostly found in factorial designs and often disappears when Age of Acquisition is controlled for or entered in a regression model (Carroll and White, 1973; Morrison et al., 1992; Barry et al., 1997, 2001; Bonin et al., 2002, but see Snodgrass and Yuditsky, 1996; Barry et al., 1997; Ellis and Morrison, 1998). Some authors attribute the effect of lexical frequency to lexical (lemma) selection (Dell, 1990; Alario et al., 2002; Navarrete et al., 2006) while others attribute it to phonological encoding (Jescheniak and Levelt, 1994; Levelt et al., 1999). Recent research suggests an effect of lexical frequency at both processing stages (Kittredge et al., 2008; Knobel et al., 2008). An ERP study by Strijkers et al. (2010) reported waveform divergences between high and low frequency words at 180 ms after picture presentation. The authors suggested that this time period corresponds to the initiation of lexical selection. Further evidence in favor of a lexical-phonological locus of the word frequency effect is provided by fMRI overt picture naming studies. Graves et al. (2007) reported a correlation between lower word frequency and activity in the left posterior superior temporal gyrus, a region previously associated with lexical-phonological processing in word production (see Price, 2012 for a review). Wilson et al. (2009) showed activation related to word frequency effect in an anatomical contiguous region (the left posterior inferior temporal gyrus).

*Image agreement* refers to the proximity between a represented object and its mental image. Participants were asked to rate on a five-point scale (from *low agreement* to *high agreement*) the agreement between a pictorial stimulus and their own mental representation of the depicted object (Alario and Ferrand, 1999). Effects of image agreement on naming latencies have been found in several studies (Barry et al., 1997, see also Alario et al., 2004). Snodgrass and Vanderwart (1980) hypothesized that this measure would affect image recognition. To our knowledge, no empirical arguments have yet come to back up this view.

*Name agreement* is a measure of the degree of association between the picture and the corresponding modal name. It is estimated by examining the number of different names participants provide for a given picture. It has been shown that when participants give many different names for a same picture (low name agreement), production latencies are longer (Lachman et al., 1974; Paivio et al., 1989; Vitkovitch and Tyrrell, 1995; Snodgrass and Yuditsky, 1996; Barry et al., 1997; Alario et al., 2004; Kan and Thompson-Schill, 2004). Name agreement does not affect object decision reaction times, suggesting that the effect of this variable on naming responses occurs during lexical retrieval, and/or during phonological encoding (Johnson et al., 1996; Alario et al., 2004). In line with this hypothesis, Cheng et al. (2010) reported an effect of Name Agreement on ERPs in a silent picture naming task at 290 ms from picture onset, a time window usually associated with phonological encoding processes. Note that these authors also found an early influence of Name agreement in the P1 time window (around 120 ms after picture onset). According to the authors, this early influence could reflect the enhanced recruitment of visual attentional resources for pictures with low relative to high name agreement.

*Age of acquisition* (AoA) refers to the age at which a given word is learnt. Numerous studies have shown that words acquired earlier are named faster, and that different brain activations underlie the processing of early and late acquired words (see for instance Hernandez and Fiebach, 2006; for brain correlates of AoA effects in word reading). The effect appears to be similar with subjective estimates of AoA (e.g., Morrison and Ellis, 1995; Chalard and Bonin, 2006) and with objective measures taken from corpora of child speech (Morrison et al., 1997; Ellis and Morrison, 1998). Reliable effects have also been found when frequency is controlled for (Barry et al., 2001). Some authors have ascribed AoA effects to lexical-semantic encoding stages, reporting independent AoA effects in tasks which did not necessarily involve access to the word form, but rather lexical-semantic processing, such as semantic blocking in picture naming and semantic categorization (Belke et al., 2005; Johnston and Barry, 2005). Other studies converge toward a lexical-phonological locus of AoA. Morrison et al. (1992) found that AoA was a significant predictor of picture naming speed but did not affect semantic categorization, suggesting that the effect originates in the retrieval and articulation of object names. Morrison and Ellis (1995) reported AoA effects in lexical decision and in immediate but not delayed picture naming, concluding for its implication in retrieval of the word form. Recent ERP data (Laganaro and Perret, 2011; Laganaro et al., 2012) revealed that AoA modulated ERPs recorded during picture naming in a relatively late time window compatible with retrieval of the word form.

### Word length

Studies on the influence of word length on picture naming latencies have reported mixed outcomes (see Cuetos et al., 1999; Santiago et al., 2000; Roelofs, 2002 for shorter latencies for shorter words and Snodgrass and Yuditsky, 1996; Bachoud-Lévi et al., 1998; Dell'Acqua et al., 2000; Damian et al., 2010 for null effects). As for the attribution of this effect, most models of word production assume that longer words should take longer to be named due to the sequential insertion of phonemes in the metrical structure during phonological encoding. Hence, if length does have an effect, we can assume it is located after lexical retrieval, most likely in late time windows associated with phonological encoding. Graves et al. (2007) reported word length effects in primary motor regions, suggesting for the involvement of such factor during phonetic encoding and implementation of the articulatory routine.

More recently, other variables such as phonological neighborhood density (Vitevitch, 2002; Vitevitch and Sommers, 2003) and phonotactic probability (Vitevitch et al., 2004) have been shown to affect speech production. The precise locus of the effect of these variables is still controversial. However, as they were not considered or balanced across conditions in previous studies, they may have influenced the outcome through their correlation with other predictors.

Building on previous chronometric and ERP studies, the present research aims at determining the time course of picture naming latencies predictors, this time course being instantiated in specific and experimentally defined periods of electrical stability (topographic maps or event-related brain potential microstates). Topographic analysis is a reference-independent measure of electrical potential variations in the brain. The main theoretical assumption of this approach is that different topographic maps are generated by different cerebral sources and supposedly different cognitive processes (Michel et al., 2009). These analyses do not only provide an insight into when processes differ but also into “how they differ in terms of likely underlying neurophysiologic mechanisms” (Murray et al., 2008, p. 249). Another characteristic of topographic analysis is that it is not affected by the choice of a reference electrode (see Michel et al., 2009).

The topographic analysis entails a spatio-temporal segmentation of the ERPs in periods of electrophysiological stability (topographic maps); crucially for the purposes of this study, topographic analysis provides information regarding the precise time course of each stable electric field configuration, with no need for a priori focus on specific time windows. The application of this analysis to stimulus- and response-aligned ERPs adapted to each individual production latency (following Laganaro and Perret, 2011), allows us to cover the entire encoding process from picture onset to articulation and to capture those encoding processes that are truncated when fixed stimulus-aligned ERP time-windows are analyzed. The standard procedure in topographic analysis requires a time point by time point computation of the spatial correlation between the template maps observed in the group-average ERP in *n* different experimental conditions and individual ERP data. This methodology allows one to investigate for instance the association between template maps and particular experimental conditions (e.g., Murray et al., 2008) or to look for differences in the duration of periods of stable electrophysiological stability across experimental conditions (e.g., Laganaro et al., 2012). This in turn allows one to draw conclusions about the dynamics of the cognitive processes involved in these different conditions. In the present study, we will conduct mixed-effects regression analyses to determine the influence of multiple variables on picture naming latencies and ERPs. Differently from the traditional approach in topographic analysis, here the time point by time point comparison of the template topographic maps identified in the grand-average ERPs will be conducted on a trial by trial basis rather than in subject-averaged ERPs. This will allow a thorough verification at the level of single trial activity—corresponding to different linguistic stimuli—of the template maps issued from the spatio-temporal segmentation of the group-averaged ERP and the analysis of the effects of a set of linguistic properties related to the words, i.e., to the trials.

This approach has several advantages. Firstly, given that many variables can be considered at once, it provides information on the time course of the whole production process rather than on a single processing step. This is important, as estimates of the time course of word production can be more precise if extracted from a single experiment rather than from different studies. Secondly, the inclusion of many variables also ensures that a given variable is significant over and above the effect of other variables. Thirdly, this methodological approach enables the use of continuous variables rather than categorical ones. As underlined by several researchers (e.g., Balling, 2008; Baayen, 2010), factorial designs have many disadvantages when compared to regression designs, including loss of power and influence of confounding variables.

An example of the application of the trial by trial approach presented here can be found in Bürki and Laganaro (2014), where it has been applied as a statistical validation tool for the comparison of an experimental condition involving multi-word production in comparison to bare noun production in a picture naming task.

## Methods

### Participants

Thirty one undergraduate students (7 men), recruited at the University of Geneva participated in the study. They were all native French speakers, aged between 18 and 36 (mean = 24). They were all right-handed as determined by the Edinburgh Handedness Scale (Oldfield, 1971). Twenty-one participants performed the picture naming task in the framework of the present study and 10 subjects were selected among the participants with the highest rate of uncontaminated EEG epochs from a previous study using the same material and procedure (Laganaro et al., 2012).

The participants gave their informed consent—approved by the local ethics committee—and were paid for their participation.

### Material

The stimuli were 120 words and their corresponding black and white line drawings from two French databases (Alario and Ferrand, 1999; Bonin et al., 2003), from which a subset of 100 items was selected. The retained 100 words were those with the highest rate of correct responses (global accuracy: 95.2%) and with a minimum of 20 uncontaminated EEG epochs (see pre-analyses). The stimuli characteristics are provided in Supplementary Material.

### Procedure

Participants were tested individually in a soundproof dark room. They sat 60 cm in front of the computer screen. Pictures were presented in constant size of 9.5 × 9.5 cm (approximately 4.52° of visual angle) on a gray screen. Before the experiment, participants were familiarized with all the pictures and their corresponding names on a paper sheet. An experimental trial had the following structure: a fixation sign was presented for 500 ms followed by the presentation of a picture on the screen for 2000 ms. Participants were asked to name the picture as quickly as possible. A 2000 ms blank screen was displayed before the next trial. Items were presented in different pseudo-random orders for each participant, which were controlled to avoid for semantically or phonologically related items to appear in direct succession. The experiment lasted about 15 min and started with four warming-up filler trials.

### Behavioral analyses

Each spoken response was first checked for accuracy. No-responses, wrong responses (i.e., the participant produced a different name than the one expected), hesitations and/or auto-corrections during articulation were counted as errors. A total of 50 responses (1.6% of the total) were excluded.

Response times (defined as the time between the onset of picture presentation and the onset of the verbal response) were precisely defined on the basis of the spoken responses' spectrogram. We further excluded the 62 responses (2% of the total) with a response time below 500 ms or above 1500 ms.

### EEG recording and pre-analysis

A high density EEG (128 channels covering the scalp) was recorded, using the Active-Two Biosemi system (Biosemi V.O.F. Amsterdam, Netherlands). Signals were sampled at 512 Hz and the band-pass filters were set between 0.16 and 100 Hz. Post-acquisition analyses were conducted with the Cartool Software (Brunet et al., 2011). Stimulus-aligned epochs—from picture onset to 450 ms—and response-aligned epochs—covering from −550 to 100 ms before the onset of each single verbal response- were extracted and band-pass filtered between 0.2 and 30 Hz. All epochs with out-of-range amplitudes (±100 μV) were excluded. The remaining epochs were visually checked for undetected artifacts caused by eye blinking or muscular activity. Contaminated epochs were excluded from the averaging process. Bad channels were interpolated on each epoch following a 3D spline interpolation method. Only epochs for which both stimulus-aligned and response-aligned ERPs were available were retained. Stimulus and response-aligned ERPs were merged together on the basis of each trial's reaction times and the overlapping ERP from the response-aligned signal was removed. This procedure is designed to obtain an ERP covering the whole time window of encoding, from picture onset to 100 ms before the initiation of articulation (see Laganaro and Perret, 2011; Laganaro et al., 2012 for further applications). It was applied to epochs averaged across subjects (*N* = 31) and to single epochs (single trials, *N* = 2693).

### ERP analysis

A topographic pattern analysis was carried out. Topographic analysis allows compressing variability of ERPs with a procedure called “spatio-temporal segmentation” in a series of template maps, which summarize and explain at best the data (usually the grand-average). This spatio-temporal segmentation was applied to the subject-averaged data using a Topographic Atomize & Agglomerate Hierarchical Clustering (Pascual-Marqui et al., 1995; Murray et al., 2008). In order to exclude short periods of topographic instability, a given stable ERP topography had to be present for at least 20 ms to be retained. A combination of cross-validation and Krzanovski-Lai criteria was adopted to select the optimal number of template maps. The Krzanovski-Lai criterion is based on the analysis of the curvature of the dispersion curve (W), which represents a quality measure of the segmentation. The KL value, representing a relative measure of such curvature, usually reaches the peak in correspondence with the optimal clustering (Murray et al., 2008). We then compared the template maps obtained in the segmentation of the group-averaged ERPs with each individual ERP and with the single trial evoked potentials, this procedure is called “fitting.” In the fitting procedure, each time point of each individual ERP is labeled on the basis of the spatial correlation it bears with one of the template maps issued from the segmentation of the grand-average. A set of fitting time-windows is determined, based on the results of the group-averaged segmentation; the template maps included in such time windows are then fitted back in the same time window of each subject and single trial ERP. The procedure is therefore temporally constrained and requires at least two template maps to be included in a particular time window. This procedure provides information on the presence of each stable topographic configuration time point per time point and therefore also on their duration. Statistical analyses are then carried out on these two measures.

### Statistical analyses and selection of independent variables

Behavioral and EEG responses were analyzed by means of mixed-effects regression models (e.g., Goldstein, 1987, 1995; Baayen et al., 2008). All statistical analyses were conducted with the statistical software R (R Development Core Team, 2007) and mixed-effects models were computed with the packages lmerTest (Kuznetsova et al., 2013) and lme4 (Bates and Sarkar, 2007). Statistical analyses on behavioral responses aimed at determining the predictors of picture naming latencies (i.e., time between onset of picture and onset of articulation) in our dataset. Statistical analyses on ERPs aimed at determining the predictors of the duration of the stable topographic maps defined by the spatio-temporal analysis described above. Mixed effects regression models were thus run separately for each topographic map, with the duration of the map as the dependent variable.

In each regression model, participants and items were entered as crossed random effects. The same set of variables was entered as fixed effects, always in the same order: the Levensthein phonological distance measure [i.e., mean Levensthein distance (LD) from the stimulus to its 20 closest neighbors, the LD between two words being defined as the minimum number of insertions, deletions or substitutions required to generate one word from the other, see Yarkoni et al., 2008] as a measure of phonological neighborhood density. Positional segment frequency and Positional diphone frequency (i.e., sum of log frequencies of all words that contain a given segment or diphone in a given position, divided by the log frequency of all words with a segment/diphone in this position) as measures of phonotactic probability (Vitevitch and Luce, 2004); the logarithm of *lexical frequency (LexF)*, as given for Movies and Books in the French “Lexique” database (New et al., 2004, 2007); *concept familiarity (CFam);* the v*isual complexity of the pictures (VCom), Image agreement* (*IAgr*), *Name agreement (NAgr*, i.e., the percentage of participants who produced the modal name, and the H measure, see Snodgrass and Vanderwart, 1980 for details), and *Age of acquisition* (*AoA*). Measures accounting for the five last variables were taken from either Bonin et al. (2003) or Alario and Ferrand (1999) databases (these two databases provide similar measures for different sets of pictures).

For each dependent variable, we also conducted a second model in which we introduced in addition the number of syllables as a fixed effect and removed the Levenshtein phonological distance measure and phonotactic probability. This was done because number of syllables and the other two measures were correlated above 0.6. Given that these latter models were always highly similar to the first and that number of syllables never reached significance, we only report the statistical values of the models with the Levenshtein phonological measure distance and phonotactic probability, without the number of syllables.

When a given predictor could be represented by more than one measure, we examined the influence of each of these measures in separate models. For instance, lexical frequency can be measured by counting the number of occurrence of a given word in a collection of books (written lexical frequency), or in movies subtitles (spoken lexical frequency). In French, the two measures are available. We thus conducted two statistical models, one with written frequency and another with spoken frequency. This was done to ensure that the absence of an effect for a given variable was not due to the selection of the wrong measure and favor the use of measures that best accounted for our dataset.

Items and participants were entered in the model as random effects. Unless otherwise stated, all the effects we report as significant stem from models with a random slope allowing for these effects to differ among participants. Following Baayen (2008) each model was fitted twice, the second time without the residuals of the regression model larger than 2.5 times the standard deviation. Results with and without the residual outliers did not differ and the results we report stem from models without these outliers. Alpha was set to 0.05 in the response time analysis; in the ERP analyses, where five different analyses were conducted, a Bonferroni correction was applied to adjust for multiple testing (alpha set to 0.01). For each analysis, we further report the marginal (associated with the fixed effects) and conditional (associated with the fixed plus the random effects of the model) R squares (Nakagawa and Schielzeth, 2013). We also checked that there was no potentially harmful multicollinearity in our models (redundancy tests). This was never the case; all models had tolerance values above 0.5.

## Results

### Behavioral results (production latencies)

The dataset considered in the analyses contained the 2693 data points for which participants had produced a correct response and whose epoch was included in the ERP analysis. The mean production latency was 805 ms (*SD* = 181 ms).

Results revealed main effects of age of acquisition, name agreement, and image agreement. Production latencies increased with age of acquisition (β = 40.61, *t* = 3.36, *p* < 0.01) and decreased with higher name agreement (β = −3.38, *t* = −4.87, *p* < 0.0001) and image agreement (β = −22.24, *t* = −3.17, *p* < 0.01) values. None of the other predictors was significant. Statistical values for all predictors are presented in Table 1. The marginal and conditional R squares for this model were respectively of 7 and 52%. Note that the statistical model did not converge until we removed the random slope allowing for the effect of name agreement to vary amongst participants.

**Table 1:** **Summary of the mixed effects regression model for the response latencies**.

	**β**	***t***	***p***
Phonological Levenshtein distance	−24.24	−1.74	>0.08
Positional segment frequency	12.42	0.056	>0.9
Lexical frequency	−2.25	−0.70	>0.4
Familiarity	−1.19	0.32	>0.7
Visual complexity	−6.14	−0.72	>0.4
Image agreement	−22.24	−3.17	<0.01
Name agreement	−3.38	−4.87	<0.0001
Age of acquisition	40.61	3.36	<0.01

### ERPs

**Figure 2:**
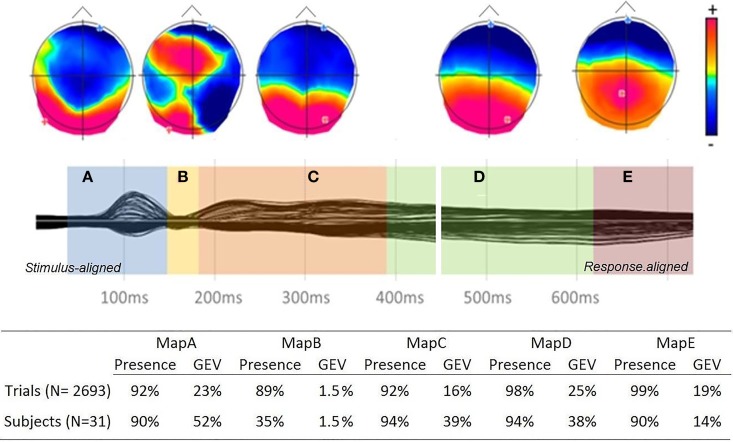
**Top:** Grand-average ERPs (128 electrodes) from onset to 100 ms before articulation and temporal distribution of the topographic maps revealed by the spatio-temporal segmentation, with map templates for the six stable topographies (positive values in red and negative values in blue with display of maximal and minimal scalp field potentials). **Bottom:** Presence and GEV of each map template in the fitting in the single trials and subject ERPs.

The spatio-temporal segmentation of the grand average from 50 ms after picture onset to 100 ms before articulation onset yielded 5 different topographic patterns, which accounted for 95.88% of the overall variance in the data (see Figure 2). Three time windows were chosen for the fitting procedure, based on the result of the segmentation on the group-average and in order to include at least two map templates in each period: from 50 to 180 ms, from 180 to 460 ms and from 460 to 100 ms before articulation. The fitting time-windows were set within rather than at the end of the time-periods of stable electrophysiological activity (topographic maps) to account for between subject and trial variability: map templates crossing the fitting borders were entered in the two consecutive fitting periods (maps “A” and “B” in the first fitting period, “B,” “C,” and “D” in the second period, “D” and “E” in last period). In order to ensure that the five topographic maps were not driven by random noise in the trial by trial data, we first performed a topographic consistency test (TCT, Koenig and Melie-García, 2010) on the trial ERPs from a subset of randomly selected items. This analysis revealed that periods of consistent topographic patterns across single trials extended from ~70 ms to the end of the analyzed period (100 ms before articulation), with the exception of a short period of topographic inconsistency from ~150 to ~180 ms in all examined items (see Supplementary Material).

A computation of Map presence was then performed separately in ERP trials and subjects. Information on map presence is obtained during the fitting procedure. Each time point of each individual trial or subject-averaged ERP is labeled accordingly to the template map with which it shares the highest spatial correlation. A measure of map presence can therefore be obtained by calculating the ratio between the number of trials in which the map was found and the total number of trials (or sample).

Results are summarized in Figure 2. To ensure that the topographic template maps issued from the spatio-temporal segmentation of the group-averaged ERP were sufficiently representative of the single trials ERPs, the Global Explained Variance (GEV) of each template map was calculated in subjects and single trials. The GEV is a measure informing on how extensively a given template map describes the variance of the considered dataset (e.g., Murray et al., 2008). Results of presence and GEV for each map in the fitting in both subjects and trials are summarized in Figure 2. The percentage of map presence is similar or higher in the single trials than in the subjects. The GEV is about 20% lower in the single trials with respect to subjects for maps A, C, and D, but it is higher in the single trials than in the subjects for the last period of topographic stability (map E).

Crucially for the single trial analysis carried out here, all maps appeared in at least 89% of the trials. The three variables found to affect production latencies were entered as fixed predictors in each regression analysis along with the other psycholinguistic variables as covariates. This ensured that the effects of some were not by-products of their correlations with other variables.

Results of the mixed effects regression model for each stable electrophysiological pattern are summarized in Table 2.

**Table 2:** **Summary of the mixed-effects regression model for the duration of periods of stable electrophysiological activity (topographic maps)**.

	**Map A: ~50–140 ms β, t, sign**	**Map B: ~140–180 ms β, t, sign**	**Map C: ~180–380 ms β, t, sign**	**Map D: ~380–620 ms β, t, sign**	**Map E: ~620–articulation β, t, sign**
VCom		−1.92, *t* = −2.85, ^**^			
CFam					
IAgr				−8.67, *t* = −2.75, ^**^	
NAgr				−1.08, *t* = −4.17, ^***^	−0.67, *t* = −4.73, ^***^
AoA				9.92, *t* = 2.48, *p* = 0.015	7.6, *t* = 3.08, ^**^

** p < 0.01;

*** *p* < 0.001.

*VCom, visual complexity; CFam, Concept Familiarity; IAgr, Image Agreement; NAgr, Name Agreement; AoA, Age of Acquisition*.

The duration of the second map (map “B”), which lasted from about 140 to 180 ms after picture onset, decreased with visual complexity (*p* < 0.01). Map D, which started around 380 ms after picture onset and lasted for about 240 ms, decreased with higher Image *Agreement* (*p* < 0.01) and *Name agreement* (*p* < 0.0001) values. There was also a marginally significant effect of the *word Age of Acquisition* (*p* = 0.015). The last stable pattern (map “E” in Figure 2), which started about 620 ms after picture onset and lasted until at least 100 ms before the onset of articulation had a longer duration for late-acquired words (*p* < 0.01) and words with low name agreement values (*p* < 0.0001). The complete statistical models are reported in Supplementary Material. Note that the model for map D did not converge with a random slope for Name Agreement, and the one for map E did not converge until the removal of the random slopes allowing for the effects of Name Agreement and Image agreement to differ among participants.

## Discussion

The aim of the present work was to gain insight into the dynamics of word production in picture naming tasks. To this end, we analyzed the effects of a set of theoretically relevant variables on response times as well as on an electrophysiological measure, namely the duration of periods of stable EEG activity (topographic maps). A multiple regression approach was implemented on trial by trial ERPs covering the entire encoding period from picture onset to 100 ms before articulation. This approach allowed us to select the variables that truly influenced response times in our dataset and to pinpoint the exact time windows at which these variables exerted their influence.

**Figure 3:**
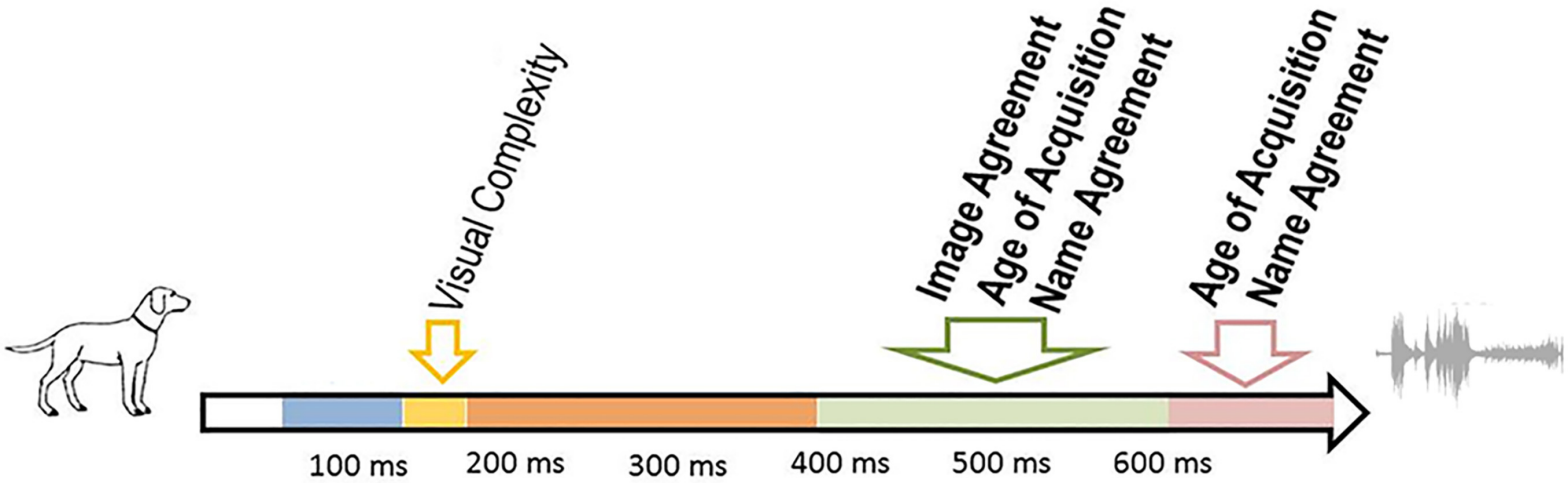
**Summary of the time course of the significant predictors in the ERP analyses**. Variables that also affected RTs are in bold.

Three out of the larger set of examined variables had robust independent effects on production latencies: *word age of acquisition, name agreement*, and *image agreement*. Overall, these results confirm previous published data on the predictors of picture naming latencies. Effects of name agreement, age of acquisition, and image agreement have indeed been reported in many studies (see Alario et al., 2004 for reviews). The five remaining variables (lexical frequency, visual complexity, familiarity, word length, phonological neighborhood, and phonotactic probability) have also been reported to affect production latencies in previous studies but much less systematically, especially when multiple regression designs were used (see Alario et al., 2004). Crucially for our purposes, the three variables that had an effect on RTs also significantly affected the duration of periods of topographic stability. The results are summarized in Figure 3. In what follows, we will discuss these results in the light of previous psycholinguistic and ERP findings.

The visual complexity of the pictures modulated ERPs but did not affect RTs. *Visual complexity* modulated the duration of the second period of topographic stability (topographic map B, from ~140 to ~180 ms). The shorter duration for more complex pictures likely reflects the fact that pictures can be recognized faster when they contain more details; this is in agreement with studies reporting shorter production latencies for more complex pictures (Szekely et al., 2005, but see Alario et al., 2004). The effect falls within the P1-N1 range, traditionally associated with visual-conceptual processing (Schendan and Kutas, 2003). Similar results have been reported by Martinovic et al. (2008, Experiment 2) who compared ERPs of pictures with high vs. low visual complexity in a gender decision task. Visual complexity did not affect response times, but modulated ERPs in the P1 range with higher amplitudes and increased evoked gamma-band activity, but also earlier peak latency for high complexity relative to low complexity pictures. The upper boundary of the visual complexity effect likely indicates that the limit of pre-linguistic processing in picture naming lays at approximately 180 ms. This result further supports the reliability of the trial by trial approach adopted here. An influence of the predictor *Visual Complexity* on the duration of the stable topographic configuration in the first 180 ms is perfectly consistent with the robust and extensive evidence associating this specific time-window with the processing of visual object information.

*Name agreement* (NAgr) modulated the duration of two stable electrophysiological patterns in the time window extending between approximately 380 after picture onset and 100 ms before articulation (Maps “D” and “E” in Figure 2). The duration of these two maps decreased with higher name agreement values. Name agreement is an objective measure of the degree of association between the picture and its modal name.

Low agreement values can have two alternative sources: either the picture is visually ambiguous, or it has several possible names. The finding that the effect of Name agreement occurs rather late in the production process suggests that in the present study, the effect results from the latter and likely takes place during the word's phonological encoding. The erroneous responses provided by the participants in Alario and Ferrand (1999) and Bonin et al. (2003) to the stimuli with low NA support this hypothesis, as the majority of errors were synonyms of the modal name. This finding is in line with previous attributions of this effect to lexical retrieval and/or phonological encoding (Johnson et al., 1996; Alario et al., 2004, see the Introduction) as well as with Cheng et al. (2010) who reported an influence of Name agreement around 290 ms after picture onset. As already noted in the Introduction, Cheng et al. also found an effect of Name Agreement at around 120 ms after picture onset, i.e., in the P1 range. Possibly, this early effect resulted from visual properties of the pictures. Given, however, that in the Cheng et al.'s study the participants performed a covert picture naming task, comparisons with the present study are not straightforward.

Word *age of acquisition* (AoA) also modulated the duration of the last period of stable electrophysiological pattern (map E) and marginally with our extremely conservative correction criteria of the duration of map D. The duration of these two topographic maps increased for late-acquired words. As reviewed in the Introduction, recent ERP studies converge toward a late locus of AoA effects (see Perret et al., 2014 for recent evidence and discussion). The temporal signature of word Age of Acquisition has been investigated by Laganaro and Perret (2011) and Perret et al. (2014) in picture naming tasks, with ERP topographic analyses. The authors found that word age of acquisition modulated ERPs at ~350–400 ms after picture presentation (for an overall response time of ~750 ms), a time window compatible with lexical-phonological encoding processes. The present results corroborate these findings. Note that according to Indefrey's (2011) estimate, phonological encoding is engaged between 275 and 450 ms after picture onset. Importantly, however, these estimates were based on mean response latencies of 600 ms. An earlier ERP investigation on the time course of word production comparing different response latencies indicated that lexical (lemma) selection can be lengthened in case of slower production speed (Laganaro et al., 2012), thus delaying phonological encoding (shifting it to the right on the temporal axis); this also seems to be the case in the present data, as mean production latencies are about 800 ms.

*Image agreement* (IA) modulated the duration of the stable topographic configuration ranging from approximately 380 to 620 ms after picture onset. Higher image agreement yielded shorter durations of map D. The concept of Image agreement was formalized by Snodgrass and Vanderwart (1980). These authors asked participants to judge the degree to which a picture would correspond to the mental object of that picture's name. Barry et al. (1997, see also Alario et al., 2004) found that the higher these scores, the shorter the naming latencies. The authors hypothesized that image agreement exerts its influence during object recognition. Accordingly, it should modulate ERPs in an early time-window associated with pre-linguistic processes. This suggestion was rather intuitive, based on the fact that IA should code the prototypicality of the picture for a given object. Our results are at odds with this interpretation, since IAgr modulated ERPs in the same time-window as AoA and NAgr, i.e., in the time window associated with lexical-phonological processes. This finding questions the association of Image Agreement with pre-linguistic processes, and rather suggests that it refers to the link between the picture and its name. Possibly, IAgr and NAgr reflect the same underlying predictor but are measured differently (as evident from the low correlation between the two measures (*r* = 0.2) and their independent effects on response times and map durations). When asked to estimate Image Agreement, participants are first presented with the name of the picture to rate, and this information likely plays a major role on their ratings. Unlike Name Agreement, however, Image Agreement measures are based on subjective estimated strengths between the picture and the concept. Raters will thus differentiate, for instance, between pictures with a single possible noun, pictures with many nouns among which one is clearly dominant, and pictures with many nouns without a clear favorite. By contrast, Name Agreement measures are based on the objective number of responses provided for a given picture. Consequently, they should not differentiate between the two first categories. It is also worth noting here that the effect of Image agreement, unlike that of NAgr and AoA, does not extend to the following map. This suggests that the mechanisms that are responsible for two consecutive effects for NAgr and AoA do not have a general character.

Interestingly, the duration of the stable electrophysiological activity in the time window ranging from about 180 to about 380 ms was not affected by any of the variables considered in our study. This period of topographic stability covers a time window which has been previously associated with lexical selection (e.g., Strijkers et al., 2010, see also Indefrey, 2011). The only variable whose influence is thought to originate at least partially during lexical selection is lexical frequency. In the present study, there was no effect of this variable on response times or on any of the periods of topographic stability whatever the lexical frequency measure (spoken or written) considered. This lack of effect cannot be due to the correlation of this variable with other predictors, which are quite low (*r* = −0.366 with image agreement, *r* = 0.283 with familiarity and *r* = −0.260 with AoA, all other correlations <0.2). Moreover, lexical frequency does not affect response latencies or the duration of map C, even when entered as the only predictor in the statistical model. In previous studies with picture naming tasks, effects of lexical frequency are reported with factorial designs, where the difference between frequency conditions is maximized (Strijkers et al., 2010) or with continuous measures of lexical frequency in large sets of items. For instance, whereas effects of lexical frequency on naming responses are reported in Alario et al. (2004) with 400 items or in Bonin et al. (2003) with 300 items (400 words), previous studies with 200 items or less did not find any effect of lexical frequency (Bonin et al., 2002; Chalard et al., 2003). In the present study, we used a continuous measure of lexical frequency, with only 100 items and the lack of frequency effect is thus in line with previous findings.

The effects of Name Agreement and of AoA on the two last successive periods of topographic stability raise several crucial points for the architecture of language production models. Although we cannot rule out that these two observations are independent (i.e., Name Agreement and Age of Acquisition may affect each of these periods of stability), their effect on the last period may be determined by the previous one, meaning that the last process started while the previous one was not completed. This issue is closely related to the question of the dynamics of planning processes in speech production. The effect of psycholinguistic variables on two consecutive periods of electrophysiological stability, likely corresponding to two periods of mental information processing (Lehmann et al., 1998; Changeux and Michel, 2004), may be interpreted as cascade activation from one mental process to the other. Hence, although the interpretation of the time-course of the sequences of periods of topographic stability was framed within the framework of serial word production models (Levelt et al., 1999; Indefrey and Levelt, 2004), the observation that some psycholinguistic variables affect two consecutive periods of stable global electric field suggest interaction (at least in terms of cascading) during these late word planning processes. In the same vein, effects limited to a single stable period (as the effect of visual complexity on map B in the present study) may indicate strictly sequential processes at other planning time-windows. The present study was not designed to explicitly address this question, but the rationale exposed here may be implemented in further investigation to determine if and when interaction is observed in word production.

In addition to documenting important theoretical issues on the time course of word production in picture naming, this research opens up new methodological prospects. So far, previous research on language production using ERPs relied on factorial designs. As underlined by several authors, factorial designs have several drawbacks, including lack of systematic control of potential confounds, and loss of statistical power. By contrast, the methodology used here does not suffer from these downsides and, as such, is particularly valuable for language studies, where the properties of the linguistic materials are salient variables. Moreover, in classical analyses, ERPs are averaged across subjects. Consequently, the statistical models do not provide information about the variance related to the items, and one may question whether their outcomes can truly be generalized across words (e.g., Barr et al., 2013). It is worth noting that several observations in our data suggest that our approach is extremely robust. Firstly, each topographic map revealed by the spatio-temporal segmentation on the grand-average ERPs was present in at least 89% of the single trials (up to 100% for some maps). Secondly, the rate of topographic map presence was comparable or higher in single trials than in subjects averaged ERPs. Moreover, the TCT analysis revealed high consistency across trials, except in the short time period ranging from ~150 to ~180 ms after picture onset. As previously advocated in other cognitive domains (Tzovara et al., 2012) a high rate of stable electrophysiological map presence licenses a trial by trial approach.

To conclude, the classical mental chronometry approach in cognitive psychology holds that any increase in response latency by a given variable reflects an underlying processing cost. The ERP analysis applied here to picture naming data allowed us to associate the cost generated by psycholinguistic variables to the duration of stable electrophysiological processes. This approach identified the time windows at which Visual Complexity, Name Agreement, Age of Acquisition and Image Agreement exert their influence and provided novel and precise information on the time course of word production processes in object picture naming.

## Conflict of interest statement

The authors declare that the research was conducted in the absence of any commercial or financial relationships that could be construed as a potential conflict of interest.
