# Supplementary material for: ERP correlates of word production predictors in picture naming: a trial by trial multiple regression analysis from stimulus onset to response
Source: Front Neurosci. 2014 Dec 4;8:390. doi: 10.3389/fnins.2014.00390 (PMC4255522; doi:10.3389/fnins.2014.00390)
Supplement: Supplementary file 1 [file DataSheet1.DOCX]

APPENDIX 1: Stimuli used in the Experiment and their properties

| Item | N of Correct responses | N of valid EEG epochs | Name agree-ment (H) | Name agree-ment (%) | Image agree-ment | Concept fami-liarity | Visual comple-xity | Age of Acqui-sition | Log Lexical freq. (movies) | Lexical freq. (Frantext) | N.of sylla-bles | Positional segment freq. | Positional diphone freq. | Phonological Levensthein distance |
| --- | --- | --- | --- | --- | --- | --- | --- | --- | --- | --- | --- | --- | --- | --- |
| *mean* | *29.64* | *26.93* | *0.16* | *93.03* | *3.63* | *3.01* | *2.99* | *2.17* | *1.1* | *20.02* | *1.79* | *0.25* | *0.02* | *1.56* |
| *SD* | *1.35* | *1.93* | *0.21* | *8.29* | *0.73* | *0.89* | *0.97* | *0.48* | *0.46* | *30.94* | *0.68* | *0.12* | *0.02* | *0.43* |
| **abeille** | 28 | 21 | 0.47 | 65 | 3.63 | 2.43 | 4.93 | 1.88 | 0.7 | 3.2 | 2 | 0.16 | 0.008 | 1.85 |
| **allumette** | 30 | 29 | 0 | 100 | 4.95 | 4.05 | 1.65 | 2.4 | 0.7 | 5.5 | 3 | 0.29 | 0.023 | 1.9 |
| **ananas** | 31 | 30 | 0 | 100 | 4.77 | 2.73 | 4.52 | 2.46 | 0.5 | 2.1 | 3 | 0.23 | 0.009 | 1.8 |
| **âne** | 31 | 25 | 0 | 100 | 4.2 | 2.07 | 3.59 | 2.08 | 1.1 | 10.8 | 1 | 0.09 | 0.003 | 1 |
| **araignée** | 31 | 28 | 0.15 | 96 | 3.03 | 2.2 | 3.21 | 2.15 | 1.1 | 8.3 | 3 | 0.31 | 0.021 | 1.95 |
| **arc** | 31 | 27 | 0 | 95 | 4.4 | 1.85 | 3.3 | 2.4 | 0.7 | 29.7 | 1 | 0.21 | 0.01 | 1 |
| **arrosoir** | 28 | 25 | 0 | 96 | 4 | 2.27 | 2.72 | 2.31 | 0.1 | 1.5 | 3 | 0.39 | 0.037 | 2.4 |
| **avion** | 30 | 27 | 0.29 | 98 | 3.93 | 2.63 | 3.17 | 1.92 | 2 | 34.7 | 2 | 0.12 | 0.008 | 1.45 |
| **bague** | 28 | 24 | 0 | 96 | 2.63 | 4 | 2.28 | 2.32 | 1.4 | 9.1 | 1 | 0.18 | 0.012 | 1 |
| **baignoire** | 30 | 27 | 0 | 100 | 4.35 | 4.4 | 3.05 | 2.4 | 1.1 | 7.2 | 2 | 0.29 | 0.03 | 1.95 |
| **balai** | 29 | 28 | 0 | 93 | 3.77 | 4.1 | 2.28 | 1.95 | 1 | 7.7 | 2 | 0.28 | 0.029 | 1 |
| **balançoire** | 30 | 27 | 0 | 98 | 3.57 | 2.47 | 1.69 | 1.84 | 0.5 | 1.8 | 3 | 0.49 | 0.046 | 2.8 |
| **banane** | 29 | 26 | 0 | 100 | 4.6 | 3.87 | 1.21 | 1.58 | 0.9 | 2.5 | 2 | 0.3 | 0.023 | 1.7 |
| **boîte** | 27 | 26 | 0.26 | 80 | 2.7 | 2.97 | 1.21 | 1.65 | 1.9 | 58.8 | 1 | 0.19 | 0.02 | 1.3 |
| **botte** | 29 | 27 | 0.4 | 96 | 2.47 | 3.73 | 2.69 | 2.04 | 0.9 | 5.6 | 1 | 0.15 | 0.003 | 1 |
| **bougie** | 29 | 27 | 0 | 91 | 4.03 | 3.6 | 2.45 | 1.96 | 0.9 | 10.7 | 2 | 0.2 | 0.01 | 1.5 |
| **briquet** | 31 | 29 | 0.61 | 100 | 4.3 | 3.95 | 2.15 | 2.3 | 1 | 7.6 | 2 | 0.28 | 0.026 | 1.25 |
| **brosse** | 28 | 25 | 0.15 | 96 | 2.67 | 4.23 | 2.69 | 1.77 | 0.9 | 11.4 | 1 | 0.21 | 0.01 | 1.7 |
| **bureau** | 28 | 25 | 0 | 72 | 3.2 | 4.6 | 2.97 | 2.65 | 2.2 | 97.8 | 2 | 0.25 | 0.015 | 1.35 |
| **cadeau** | 31 | 30 | 0 | 100 | 1.25 | 2.95 | 3.75 | 1.75 | 2 | 18.7 | 2 | 0.27 | 0.02 | 1 |
| **cage** | 30 | 27 | 0 | 93 | 3.13 | 1.8 | 4.38 | 2.27 | 1.2 | 22.2 | 1 | 0.22 | 0.018 | 1 |
| **camion** | 30 | 29 | 0 | 100 | 2.77 | 3.23 | 2.9 | 1.62 | 1.7 | 18.2 | 2 | 0.3 | 0.027 | 1.75 |
| **canapé** | 27 | 24 | 0.34 | 65 | 3.17 | 4.4 | 2.31 | 2.16 | 1.3 | 10 | 3 | 0.44 | 0.031 | 1.95 |
| **canard** | 25 | 23 | 0.29 | 93 | 3.47 | 2.5 | 2.97 | 1.85 | 1.2 | 9.6 | 2 | 0.43 | 0.043 | 1.2 |
| **canne** | 27 | 24 | 0.31 | 95 | 4.35 | 3.3 | 4.45 | 3.45 | 1 | 16.6 | 1 | 0.24 | 0.021 | 1 |
| **carotte** | 31 | 31 | 0 | 98 | 4.47 | 3.9 | 3.07 | 1.58 | 0.5 | 2.5 | 2 | 0.43 | 0.05 | 1.25 |
| **ceinture** | 31 | 30 | 0.41 | 98 | 4.23 | 4.13 | 1.93 | 2.42 | 1.3 | 20.9 | 2 | 0.31 | 0.014 | 1.55 |
| **cendrier** | 31 | 28 | 0.16 | 96 | 3.3 | 4 | 2.62 | 2.85 | 0.7 | 5.9 | 3 | 0.52 | 0.056 | 1.95 |
| **cerise** | 30 | 26 | 0.29 | 98 | 3.77 | 3.13 | 1.34 | 2 | 0.6 | 2.5 | 2 | 0.38 | 0.024 | 1.9 |
| **cerveau** | 29 | 26 | 0.15 | 98 | 3.37 | 2.8 | 3.34 | 3.12 | 1.8 | 28.2 | 2 | 0.33 | 0.036 | 1.6 |
| **chaîne** | 29 | 27 | 0.67 | 91 | 2.93 | 2.93 | 2.72 | 2.69 | 1.5 | 45.7 | 1 | 0.1 | 0.004 | 1.15 |
| **champignon** | 30 | 26 | 0 | 96 | 3.67 | 2.9 | 3 | 2.35 | 0.6 | 4.2 | 3 | 0.21 | 0.012 | 2.65 |
| **chapeau** | 30 | 29 | 0 | 100 | 2.93 | 2.83 | 2.38 | 1.62 | 1.7 | 42.5 | 2 | 0.21 | 0.014 | 1.35 |
| **chien** | 31 | 31 | 0 | 100 | 2.23 | 3.8 | 2.76 | 1.19 | 2.2 | 69.7 | 1 | 0.04 | 0.004 | 1.5 |
| **cigarette** | 29 | 26 | 0.29 | 98 | 3.93 | 4.1 | 2.17 | 2.38 | 1.6 | 40.5 | 3 | 0.48 | 0.056 | 2.8 |
| **ciseau** | 31 | 28 | 0.34 | 100 | 4.5 | 4.07 | 2.24 | 2 | 0.3 | 2.4 | 2 | 0.21 | 0.014 | 1.6 |
| **citron** | 30 | 24 | 0 | 100 | 4.83 | 3.63 | 1.72 | 1.88 | 1 | 8.1 | 2 | 0.34 | 0.033 | 1.5 |
| **cloche** | 31 | 28 | 0.15 | 96 | 3.7 | 2.1 | 3 | 2.19 | 1 | 15.1 | 1 | 0.15 | 0.006 | 1.55 |
| **collier** | 31 | 27 | 0.47 | 100 | 3.9 | 3.33 | 1.79 | 1.86 | 1.3 | 9 | 2 | 0.33 | 0.036 | 1.55 |
| **commode** | 28 | 25 | 0.15 | 76 | 3.3 | 4.27 | 2.83 | 2.96 | 0.6 | 23.5 | 2 | 0.21 | 0.022 | 1.8 |
| **crabe** | 29 | 25 | 0 | 78 | 4.13 | 2.1 | 4.1 | 2.38 | 0.8 | 5 | 1 | 0.26 | 0.026 | 1.45 |
| **cravate** | 31 | 30 | 0 | 93 | 3.8 | 3.33 | 2.66 | 2.38 | 1.2 | 15.5 | 2 | 0.41 | 0.035 | 1.9 |
| **croissant** | 30 | 29 | 0 | 100 | 4.77 | 3.77 | 2.93 | 2.04 | 0.4 | 13.7 | 2 | 0.36 | 0.033 | 1.55 |
| **cube** | 28 | 25 | 0.34 | 100 | 3.8 | 2.8 | 1.15 | 2.25 | 0.4 | 6.4 | 1 | 0.15 | 0.004 | 1.4 |
| **dent** | 31 | 27 | 0.47 | 85 | 4.2 | 2.1 | 3.6 | 2.15 | 1.2 | 9 | 1 | 0.11 | 0.002 | 1 |
| **douche** | 28 | 25 | 0.78 | 91 | 2.33 | 4.87 | 3.72 | 2.23 | 1.5 | 10.4 | 1 | 0.14 | 0.005 | 1.15 |
| **échelle** | 29 | 26 | 0 | 96 | 3.97 | 2.7 | 2.59 | 2.27 | 1.2 | 48.5 | 2 | 0.12 | 0.004 | 1.9 |
| **éléphant** | 31 | 30 | 0 | 100 | 3.9 | 1.4 | 4.55 | 2.04 | 1 | 5.7 | 3 | 0.14 | 0.007 | 1.85 |
| **escargot** | 30 | 27 | 0 | 98 | 3.77 | 2.3 | 3 | 1.88 | 0.6 | 2.4 | 3 | 0.29 | 0.028 | 2.65 |
| **flèche** | 29 | 27 | 0 | 98 | 3.83 | 1.53 | 1.93 | 2.52 | 1 | 14.1 | 1 | 0.13 | 0.009 | 1.5 |
| **fraise** | 31 | 24 | 0 | 89 | 3.03 | 3.2 | 2.76 | 1.81 | 0.8 | 2.7 | 1 | 0.2 | 0.017 | 1.4 |
| **gâteau** | 30 | 29 | 0 | 93 | 2.87 | 3.67 | 2.34 | 1.27 | 1.6 | 9.8 | 2 | 0.25 | 0.021 | 1.35 |
| **girafe** | 30 | 28 | 0 | 100 | 4.47 | 1.3 | 4.97 | 2.12 | 0.6 | 1 | 2 | 0.3 | 0.019 | 1.95 |
| **gomme** | 31 | 28 | 0.29 | 75 | 3.95 | 3.3 | 2.55 | 3.35 | 0.6 | 6.8 | 1 | 0.08 | 0.002 | 1.45 |
| **grenouille** | 29 | 27 | 0.67 | 80 | 4.17 | 1.87 | 3.69 | 1.92 | 0.8 | 6 | 2 | 0.2 | 0.012 | 1.9 |
| **guitare** | 30 | 28 | 0.15 | 100 | 4.57 | 2.9 | 3.45 | 2.5 | 1.1 | 7.5 | 2 | 0.36 | 0.031 | 1.9 |
| **jambon** | 30 | 26 | 0.57 | 75 | 2.65 | 2.5 | 3.25 | 2.7 | 1 | 6.7 | 2 | 0.09 | 0.004 | 1.75 |
| **jumelles** | 29 | 29 | 0 | 85 | 3.63 | 2.07 | 4.52 | 2.8 | 0.8 | 9 | 2 | 0.19 | 0.014 | 1.95 |
| **jupe** | 30 | 27 | 0 | 83 | 2.37 | 3.23 | 1.66 | 1.65 | 1 | 18.1 | 1 | 0.09 | 0.006 | 1.4 |
| **lapin** | 30 | 26 | 0 | 100 | 4.07 | 2.67 | 3.14 | 1.65 | 1.4 | 10.4 | 2 | 0.19 | 0.012 | 1.45 |
| **lion** | 30 | 28 | 0 | 96 | 3.53 | 1.5 | 4.17 | 1.69 | 1.2 | 16.9 | 1 | 0.05 | 0.001 | 1.1 |
| **luge** | 28 | 25 | 0.26 | 91 | 2.83 | 1.9 | 3.28 | 2.81 | 0.3 | 1 | 1 | 0.08 | 0.003 | 1.35 |
| **maïs** | 30 | 28 | 0.56 | 93 | 3.9 | 3.1 | 4.21 | 2.6 | 0.9 | 6.8 | 2 | 0.28 | 0.024 | 1.75 |
| **marteau** | 28 | 28 | 0.15 | 96 | 2.33 | 2.1 | 2.9 | 2.19 | 1.1 | 10.3 | 2 | 0.41 | 0.065 | 1.65 |
| **miroir** | 31 | 26 | 0.47 | 90 | 4.7 | 3.35 | 2.5 | 2.6 | 1.4 | 37.1 | 2 | 0.45 | 0.042 | 1.8 |
| **montagne** | 31 | 26 | 0.29 | 96 | 2.83 | 2.67 | 2.93 | 1.88 | 1.6 | 44.3 | 2 | 0.25 | 0.022 | 1.8 |
| **moto** | 30 | 26 | 0 | 93 | 3.6 | 3 | 5 | 2.23 | 1.4 | 8 | 2 | 0.22 | 0.013 | 1.15 |
| **mouton** | 29 | 27 | 0 | 67 | 2.9 | 1.83 | 3.59 | 1.65 | 0.9 | 11.1 | 2 | 0.2 | 0.009 | 1.35 |
| **niche** | 30 | 27 | 0.15 | 80 | 4.43 | 1.83 | 2.17 | 2.42 | 0.5 | 4.4 | 1 | 0.1 | 0.003 | 1.1 |
| **nid** | 30 | 26 | 0 | 96 | 3.77 | 2.17 | 4.9 | 2.23 | 1.1 | 13.1 | 1 | 0.08 | 0.001 | 1 |
| **noix** | 29 | 27 | 0 | 100 | 4 | 4 | 2.2 | 2 | 1.1 | 8.7 | 1 | 0.09 | 0.016 | 1 |
| **note** | 30 | 26 | 0.29 | 95 | 4.25 | 4.1 | 4.1 | 2.1 | 1.5 | 48.2 | 1 | 0.12 | 0.003 | 1 |
| **palmier** | 29 | 27 | 0 | 96 | 3.83 | 2.1 | 3.76 | 3.19 | 0.4 | 2.6 | 2 | 0.46 | 0.054 | 1.9 |
| **panier** | 31 | 30 | 0 | 98 | 2.63 | 2.3 | 4.59 | 1.92 | 1.2 | 16.3 | 2 | 0.38 | 0.039 | 1.6 |
| **pantalon** | 31 | 29 | 0.15 | 100 | 3.4 | 4.87 | 2.28 | 1.54 | 1.5 | 29.3 | 3 | 0.35 | 0.03 | 2 |
| **papillon** | 31 | 26 | 0 | 93 | 4.37 | 2.33 | 4.1 | 1.92 | 1 | 13 | 3 | 0.41 | 0.044 | 1.9 |
| **passoire** | 25 | 21 | 0.73 | 74 | 3.57 | 3.67 | 3.64 | 3.12 | 0.4 | 1.5 | 2 | 0.48 | 0.058 | 1.8 |
| **peigne** | 30 | 28 | 0 | 98 | 3.83 | 3.87 | 2.69 | 2 | 0.8 | 6.8 | 1 | 0.16 | 0.009 | 1 |
| **piano** | 31 | 28 | 0 | 93 | 3.8 | 3.1 | 4.72 | 2 | 1.4 | 20.6 | 2 | 0.23 | 0.007 | 1.95 |
| **plume** | 31 | 29 | 0 | 98 | 4.2 | 2.3 | 3.66 | 2.16 | 0.9 | 28.1 | 1 | 0.18 | 0.013 | 1.35 |
| **poing** | 31 | 28 | 0 | 95 | 2.9 | 3.4 | 1.2 | 1.5 | 1.2 | 26.3 | 1 | 0.13 | 0.004 | 1.35 |
| **poire** | 30 | 26 | 0 | 93 | 4.4 | 3.37 | 1.14 | 1.81 | 0.8 | 6.4 | 1 | 0.27 | 0.024 | 1.15 |
| **pont** | 29 | 24 | 0.3 | 90 | 2.3 | 3.2 | 1.45 | 2.15 | 1.7 | 61.2 | 1 | 0.14 | 0.001 | 1 |
| **prise** | 29 | 28 | 0 | 87 | 1.83 | 3.6 | 2.55 | 2.92 | 1.4 | 84.6 | 1 | 0.27 | 0.045 | 1 |
| **pyramide** | 30 | 25 | 0 | 100 | 4.5 | 1.67 | 2.34 | 3.19 | 0.8 | 5.3 | 3 | 0.49 | 0.031 | 2.7 |
| **requin** | 28 | 27 | 0 | 91 | 3.83 | 1.87 | 2.28 | 2.85 | 1 | 1.3 | 2 | 0.17 | 0.032 | 1.3 |
| **sapin** | 30 | 27 | 0.29 | 100 | 3.7 | 3.45 | 2.65 | 1.95 | 0.8 | 7.2 | 2 | 0.25 | 0.015 | 1.55 |
| **seringue** | 31 | 28 | 0.29 | 93 | 4.31 | 2.2 | 3.72 | 3.5 | 0.7 | 2.5 | 2 | 0.2 | 0.004 | 1.7 |
| **singe** | 31 | 28 | 0.43 | 93 | 3.03 | 1.4 | 3.41 | 1.8 | 1.4 | 10.4 | 1 | 0.11 | 0.003 | 1.45 |
| **ski** | 30 | 29 | 0 | 93 | 4.03 | 2.73 | 2.83 | 2.64 | 1.2 | 4.7 | 1 | 0.16 | 0.003 | 1.25 |
| **soleil** | 29 | 24 | 0 | 100 | 3.53 | 4.37 | 1.14 | 1.42 | 2.1 | 227.1 | 2 | 0.29 | 0.023 | 1.85 |
| **souris** | 29 | 27 | 0.29 | 85 | 4.27 | 2.27 | 3.38 | 1.62 | 1.4 | 26 | 2 | 0.36 | 0.035 | 1.15 |
| **tabouret** | 28 | 27 | 0 | 93 | 3.6 | 3.8 | 2.24 | 2.2 | 0.6 | 7.8 | 3 | 0.38 | 0.032 | 1.95 |
| **tambour** | 28 | 25 | 0 | 96 | 3.77 | 1.57 | 2.79 | 2.15 | 0.9 | 9.1 | 2 | 0.24 | 0.011 | 1.85 |
| **toit** | 31 | 30 | 0 | 100 | 4.35 | 3.95 | 2.85 | 2 | 1.6 | 33 | 1 | 0.13 | 0.015 | 1.05 |
| **tortue** | 31 | 26 | 0 | 100 | 4.1 | 2.03 | 3.28 | 1.92 | 0.7 | 4.4 | 2 | 0.3 | 0.036 | 1.85 |
| **train** | 30 | 27 | 0.67 | 93 | 2.87 | 3.97 | 4.69 | 1.73 | 2.4 | 161.6 | 1 | 0.16 | 0.019 | 1 |
| **vache** | 31 | 27 | 0 | 89 | 3.4 | 2.63 | 3.59 | 1.6 | 1.6 | 18.5 | 1 | 0.17 | 0.008 | 1.25 |
| **valise** | 31 | 29 | 0.43 | 98 | 2.77 | 3.9 | 3.48 | 2.23 | 1.5 | 23.8 | 2 | 0.32 | 0.033 | 1.75 |
| **vélo** | 30 | 28 | 0.41 | 89 | 4.2 | 3.37 | 4.17 | 1.8 | 1.5 | 13 | 2 | 0.21 | 0.011 | 1.65 |
| *Excluded items* | | |  |  |  |  |  |  |  |  |  |  |  |  |
| *balcon* | *26* | *<20* |  |  |  |  |  |  |  |  |  |  |  |  |
| *bocal* | *24* | *<20* |  |  |  |  |  |  |  |  |  |  |  |  |
| *brouette* | *29* | *<20* |  |  |  |  |  |  |  |  |  |  |  |  |
| *classeur* | *28* | *<20* |  |  |  |  |  |  |  |  |  |  |  |  |
| *clou* | *24* | *<20* |  |  |  |  |  |  |  |  |  |  |  |  |
| *épée* | *23* | *<20* |  |  |  |  |  |  |  |  |  |  |  |  |
| *lavabo* | *22* | *<20* |  |  |  |  |  |  |  |  |  |  |  |  |
| *parapluie* | *30* | *<20* |  |  |  |  |  |  |  |  |  |  |  |  |
| *perceuse* | *25* | *<20* |  |  |  |  |  |  |  |  |  |  |  |  |
| *pinceau* | *27* | *<20* |  |  |  |  |  |  |  |  |  |  |  |  |
| *pneu* | *26* | *<20* |  |  |  |  |  |  |  |  |  |  |  |  |
| *poivron* | *19* | *<20* |  |  |  |  |  |  |  |  |  |  |  |  |
| *portefeuille* | *22* | *<20* |  |  |  |  |  |  |  |  |  |  |  |  |
| *poumons* | *26* | *<20* |  |  |  |  |  |  |  |  |  |  |  |  |
| *robinet* | *25* | *<20* |  |  |  |  |  |  |  |  |  |  |  |  |
| *selle* | *25* | *<20* |  |  |  |  |  |  |  |  |  |  |  |  |
| *tournevis* | *28* | *<20* |  |  |  |  |  |  |  |  |  |  |  |  |
| *tracteur* | *28* | *<20* |  |  |  |  |  |  |  |  |  |  |  |  |
| *tunnel* | *29* | *<20* |  |  |  |  |  |  |  |  |  |  |  |  |
| *vase* | *25* | *<20* |  |  |  |  |  |  |  |  |  |  |  |  |

APPENDIX 2. Results of the topographic consistency test across single trials are displayed for 5 items (a): ananas – pineapple-; (b): cloche – bell-; (c) cravate –tie-; (d): champignon -mushroom; (e): valise – suitcase) for stimulus- and response-aligned ERPs with the GFP amplitude displayed on the y-axes and time on the x-axes. The grey bars represent periods of topographic inconsistency.


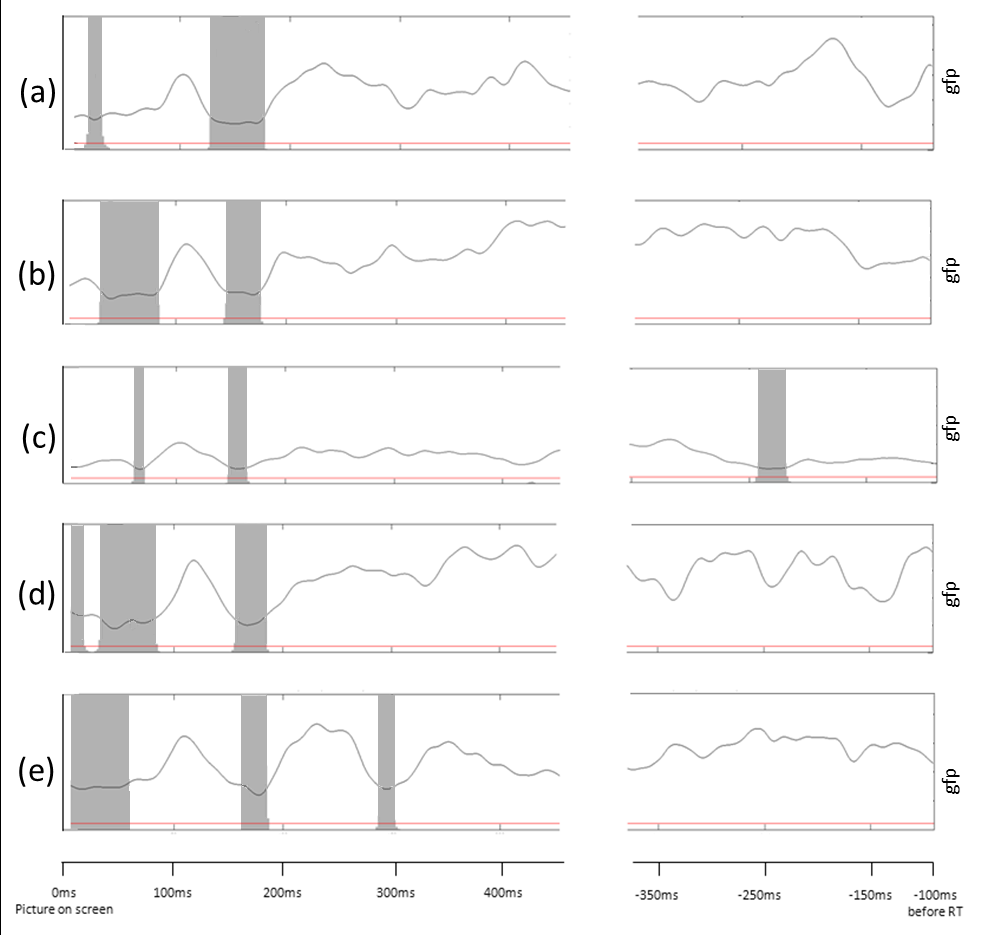


APPENDIX 3. Results of the mixed effects models for the duration of map A to E.

Map A

| **Predictor** | | **β** | | **t** | | **P value** | |
| --- | --- | --- | --- | --- | --- | --- | --- |
| Phonological Levenshtein distance | | 0.94 | | 1.29 | | > 0.2 | |
| Positional segment frequency | | -0.89 | | -0.33 | | > 0.7 | |
| Lexical frequency | | 0.18 | | 0.68 | | > 0.4 | |
| Familiarity | | 0.76 | | 2.18 | | > 0.03 | |
| Visual complexity | | -0.37 | | -1.27 | | > 0.2 | |
| Image agreement | | 0.70 | | 1.77 | | > 0.08 | |
| Name agreement | | -0.01 | | -0.16 | | > 0.8 | |
| Age of acquisition | | 0.22 | | 0.38 | | > 0.7 | |

Marginal R^2^ = 0.9%; Conditional R^2^= 24%

Map B

| **Predictor** | | **β** | | **t** | | **P value** | |
| --- | --- | --- | --- | --- | --- | --- | --- |
| Phonological Levenshtein distance | | 3.18 | | 1.66 | | > 0.1 | |
| Positional segment frequency | | -9.94 | | -1.43 | | > 0.1 | |
| Lexical frequency | | 0.45 | | 0.57 | | > 0.5 | |
| Familiarity | | -0.49 | | -0.92 | | > 0.3 | |
| **Visual complexity** | | **-1.92** | | **-2.85** | | **< 0.01** | |
| Image agreement | | -0.55 | | -0.62 | | > 0.5 | |
| Name agreement | | -0.04 | | -0.36 | | > 0.7 | |
| Age of acquisition | | 1.15 | | 0.94 | | > 0.3 | |

Marginal R^2^ = 0.5%; Conditional R^2^= 29%

Map C

| **Predictor** | | **β** | | **t** | | **P value** | |
| --- | --- | --- | --- | --- | --- | --- | --- |
| Phonological Levenshtein distance | | -0.63 | | -0.35 | | > 0.7 | |
| Positional segment frequency | | -5.45 | | -0.64 | | > 0.5 | |
| Lexical frequency | | 0.11 | | 0.079 | | > 0.9 | |
| Familiarity | | 0.14 | | 0.002 | | > 0.9 | |
| Visual complexity | | 1.37 | | 1.48 | | > 0.1 | |
| Image agreement | | -0.76 | | -0.65 | | > 0.5 | |
| Name agreement | | 0.12 | | 1.119 | | > 0.2 | |
| Age of acquisition | | 0.37 | | 0.11 | | > 0.9 | |

Marginal R^2^ = 0.3%; Conditional R^2^= 30%

Map D

| **Predictor** | | **β** | | **t** | | **P value** | |
| --- | --- | --- | --- | --- | --- | --- | --- |
| Phonological Levenshtein distance | | -10.14 | | -1.61 | | > 0.1 | |
| Positional segment frequency | | 29.60 | | 1.3 | | > 0.1 | |
| Lexical frequency | | -1.33 | | -0.75 | | > 0.4 | |
| Familiarity | | -0.06 | | -0.094 | | > 0.9 | |
| Visual complexity | | 2.10 | | 0.52 | | > 0.6 | |
| **Image agreement** | | **-8.67** | | **-2.75** | | **< 0.01** | |
| **Name agreement** | | **-1.08** | | **-4.17** | | **< 0.0001** | |
| Age of acquisition | | 9.92 | | 2.48 | | = 0.015 | |

Marginal R^2^ = 3%; Conditional R^2^= 32%

Map E

| **Predictor** | | **β** | | **t** | | **P value** | |
| --- | --- | --- | --- | --- | --- | --- | --- |
| Phonological Levenshtein distance | | -5.73 | | -1.98 | | > 0.05 | |
| Positional segment frequency | | 7.76 | | 0.66 | | > 0.5 | |
| Lexical frequency | | -0.85 | | -0.90 | | > 0.3 | |
| Familiarity | | 0.58 | | 0.32 | | > 0.7 | |
| Visual complexity | | -2.98 | | -2.20 | | > 0.03 | |
| Image agreement | | -3.74 | | -2.12 | | > 0.03 | |
| **Name agreement** | | **-0.67** | | **-4.73** | | **< 0.0001** | |
| **Age of acquisition** | | **7.64** | | **3.08** | | **< 0.01** | |

Marginal R^2^ = 4%; Conditional R^2^= 32%
